# Hematopoietic Transcription Factor RUNX1 is Essential for Promoting Macrophage–Myofibroblast Transition in Non‐Small‐Cell Lung Carcinoma

**DOI:** 10.1002/advs.202302203

**Published:** 2023-11-15

**Authors:** Philip Chiu‐Tsun Tang, Max Kam‐Kwan Chan, Jeff Yat‐Fai Chung, Alex Siu‐Wing Chan, Dongmei Zhang, Chunjie Li, Kam‐Tong Leung, Calvin Sze‐Hang Ng, Yi Wu, Ka‐Fai To, Hui‐Yao Lan, Patrick Ming‐Kuen Tang

**Affiliations:** ^1^ Department of Anatomical and Cellular Pathology State Key Laboratory of Translational Oncology The Chinese University of Hong Kong Shatin 999077 Hong Kong; ^2^ Department of Applied Social Sciences The Hong Kong Polytechnic University Hunghom 999077 Hong Kong; ^3^ College of Pharmacy Jinan University Guangzhou 510632 China; ^4^ Department of Head and Neck Oncology West China Hospital of Stomatology Sichuan University Chengdu Sichuan 610041 China; ^5^ Department of Paediatrics The Chinese University of Hong Kong Shatin 999077 Hong Kong; ^6^ Department of Surgery The Chinese University of Hong Kong Shatin 999077 Hong Kong; ^7^ MOE Key Laboratory of Environment and Genes Related to Diseases School of Basic Medical Sciences Xi'an Jiaotong University Xi'an 710061 China; ^8^ Department of Medicine and Therapeutics Li Ka Shing Institute of Health Sciences The Chinese University of Hong Kong Shatin 999077 Hong Kong

**Keywords:** cancer‐associated fibroblasts(CAF), macrophage‐myofibroblast transition (MMT), Runx1, Smad3, tumor‐associated macrophages (TAM)

## Abstract

Macrophage‐myofibroblast transition (MMT) is a newly discovered pathway for mass production of pro‐tumoral cancer‐associated fibroblasts (CAFs) in non‐small cell lung carcinoma (NSCLC) in a TGF‐β1/Smad3 dependent manner. Better understanding its regulatory signaling in tumor microenvironment (TME) may identify druggable target for the development of precision medicine. Here, by dissecting the transcriptome dynamics of tumor‐associated macrophage at single‐cell resolution, a crucial role of a hematopoietic transcription factor Runx1 in MMT formation is revealed. Surprisingly, integrative bioinformatic analysis uncovers Runx1 as a key regulator in the downstream of MMT‐specific TGF‐β1/Smad3 signaling. Stromal Runx1 level positively correlates with the MMT‐derived CAF abundance and mortality in NSCLC patients. Mechanistically, macrophage‐specific Runx1 promotes the transcription of genes related to CAF signatures in MMT cells at genomic level. Importantly, macrophage‐specific genetic deletion and systemic pharmacological inhibition of TGF‐β1/Smad3/Runx1 signaling effectively prevent MMT‐driven CAF and tumor formation in vitro and in vivo, representing a potential therapeutic target for clinical NSCLC.

## Graphical abstract

Macrophage–myofibroblast transition (MMT) is an important source of cancer‐associated fibroblasts (CAFs) in non‐small cell lung carcinoma (NSCLC). Here, the hematopoietic transcription factor Runx1 is discovered as a key regulator of MMT in cancer patients. Macrophage‐specific and systemic inhibition of Runx1 effectively blocks MMT‐driven tumor formation in vivo, representing a druggable therapeutic target to eliminate tumor‐promoting CAFs in NSCLC patients.

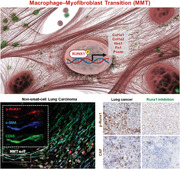

## 1 Introduction

Lung carcinoma is a major cause of death worldwide. There were 5422 new cases of lung cancer in 2020 and ranked as the top cause of cancer deaths in 2020 in Hong Kong (Hong Kong Cancer Registry). Surgery, chemotherapy, and radiotherapy have been the mainstays of treatment for decades. However, outcomes are still unsatisfactory due to side effects, metastasis, and drug resistance.^[^ ^1^ ^]^ Cancer cells are heterogeneous, versatile, and adaptable, leading to primary and secondary resistance.^[^ ^2^ ^]^ Therapies that target tumor microenvironment (TME) show promise as cancer growth, invasion, and metastasis rely on stromal conditions.^[^ ^3^ ^]^ Unexpectedly, only less than 30% of non‐small‐cell lung carcinoma (NSCLC) patients respond to the latest programmed cell death protein‐1 receptor (PD‐1) and PD‐ligand‐1 (PD‐L1) targeted therapy.^[^ ^4^ ^]^ Better understanding the TME would discover novel strategies to improve therapeutic outcome of NSCLC immunotherapy.

Cancer‐associated fibroblasts (CAFs) are a key component of TME with diverse functions for accelerating tumor progression, including matrix deposition and remodeling, stimulating proliferation and drug resistance of cells, and educating infiltrating leukocytes. CAFs are heterogeneous populations that could be arisen from number of sources,^[^ ^5^ ^]^ including local resident fibroblasts,^[^ ^6^ ^]^ epithelial cells,^[^ ^7^ ^]^ endothelial cells,^[^ ^8^ ^]^ and bone marrow‐derived myofibroblasts.^[^ ^9^ ^]^ By the most advanced single‐cell RNA‐sequencing analysis (scRNA‐seq), we discovered CAFs could be originated from bone marrow‐derived macrophages (BMDMs) via Macrophage‐myofibroblast transition (MMT) during chronic inflammation in a transforming growth factor beta1 (TGF‐β1) dependent manner. MMT is detected in TME via the co‐expression of macrophages (Cluster of Differentiation 68, CD68) and CAFs (α‐Smooth muscle actin, α‐SMA) markers. Importantly, MMT is a major source of pathogenic CAF, which constituted over half of CAF population in human NSCLC, and dramatically accelerated tumor growth in experimental lung carcinoma models. MMT may represent a potential therapeutic target for preventing the formation of protumoral CAF in NSCLC.

We recently demonstrated that Smad3 is essential for the TME‐mediated cancer progression, which largely improved the cancer host survival rate from 50% to be 100% in an experimental Lewis lung carcinoma model (LLC).^[^ ^10^, ^11^ ^]^ Nevertheless, systematic inhibition of Smad3 would affect host T cell immunity,^[^ ^12^, ^13^ ^]^ which can be avoided by directly targeting the Smad3 downstream signalling.^[^ ^14^, ^15^, ^16^ ^]^ Of note, TME of LLC (LLC‐TME) is resistant to the PD‐L1 targeted therapy.^[^ ^17^ ^]^ Our studies have identified the MMT process and tumor growth of lung carcinoma were tightly regulated by Smad3. However, targeting Smad3 systemically may also impair T‐cells' anticancer immunity. Thus, better understanding of the Smad3 downstream signaling in the TME would discover novel therapeutic targets for enhancing the therapeutic efficiency of NSCLC treatment by combination with a MMT precision therapy.

In this study, by dissecting the MMT signaling in lung cancer with our macrophage‐lineage specificscRNA‐seq dataset^[^ ^18^ ^]^ and unbiased bioinformatics, we unexpectedly discovered a hematopoietic transcription factor Runx1^[^ ^19^ ^]^ is highly expressed in the MMT cells (MMTs) and serves as a key downstream regulator in the macrophage‐specific TGF‐β1/Smad3 signaling for MMT initiation. We detected that Runx1 is highly expressed in the MMTs of NSCLC but absent in normal lung tissues. Cancer Genome Atlas database (TCGA) study^[^ ^20^ ^]^ also found its association with mortality and CAF abundance in NSCLC patients. Importantly, both macrophage‐specific and systemic inhibition of Runx1 effectively blocked the tumorigenesis of lung cancer in mice; suggesting Runx1 may represent a druggable target for preventing the formation of MMT‐driven tumor‐promoting CAFs in NSCLC.

## 2 Results

### 2.1 Stromal Runx1 Associates with CAF Formation in NSCLC

**Figure 1:**
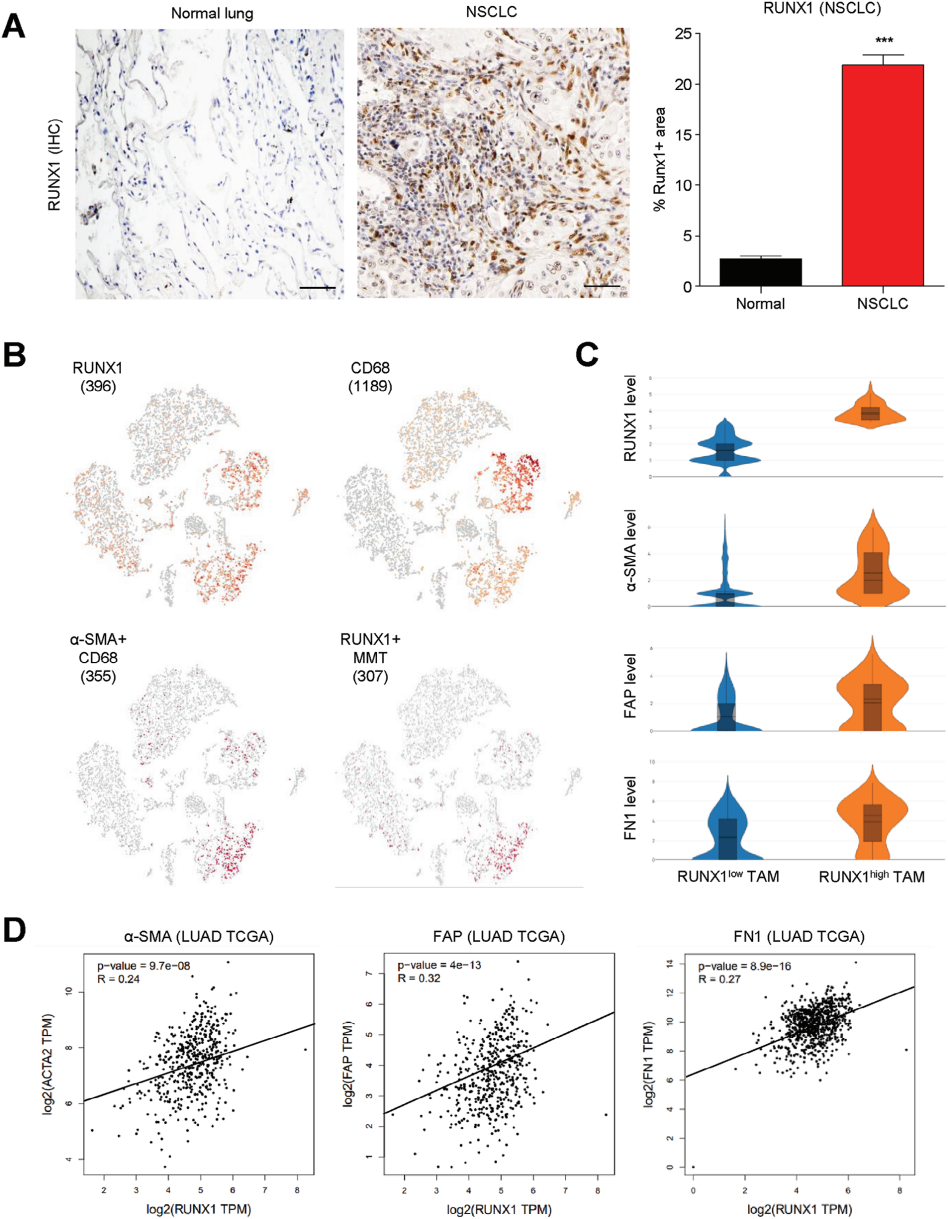
Stromal Runx1 expression correlates with CAF formation in NSCLC. A) Runx1 expression is significantly up‐regulated in tumor‐infiltrating cells of NSCLC compared to normal lung tissue (*n* = 6, ^***^ *P* < 0.001 vs normal lung). B) scRNA‐seq analysis confirms high Runx1 expression in MMTs of human NSCLC. C) CAF signatures (α‐SMA, FAP, FN1) are markedly upregulated in Runx1^high^ MMTs. D) A positive correlation between Runx1 and CAF markers is detected in the LUAD TCGA cohort, suggesting the previously unreported importance of Runx1 in MMT‐driven CAF formation. Scale bar, 50 µm.

Better understanding the molecular mechanism of MMT may discover new therapeutic target for NSCLC. Surprisingly, by conducting unbiased bioinformatics with our macrophage lineage cells specific scRNA‐seq dataset, we detected a hematopoietic transcription factor Runx1 was highly expressed in the MMTs of LLC‐tumor in vivo (Figure S1, Supporting Information). As the role of Runx1 in MMT regulation is still unknown, we examined its expression level in human NSCLC. We found that Runx1 was dramatically up‐regulated in the tumor‐infiltrating cells of NSCLC in contrast to its low expression in the normal lung tissues (**Figure** **1**A). By scRNA‐seq analysis with a public NSCLC dataset, we confirmed that Runx1 was highly expressed in the human MMTs (α‐SMA^+^CD68^+^, Figure 1B), where CAF signatures (α‐SMA, Fibroblast activation protein (FAP), Fibronectin 1 (FN1)) were markedly up‐regulated in the Runx1^high^ MMTs (Figure 1C). In line with the notion, we detected a positive correlation between Runx1 and CAF markers in the cohort study of Cancer Genome Atlas Lung Adenocarcinoma Collection (LUAD TCGA, Figure 1D), revealing an unreported importance of Runx1 in the MMT‐driven CAF formation.

### 2.2 Runx1 Correlates with MMT Development in TME

**Figure 2:**
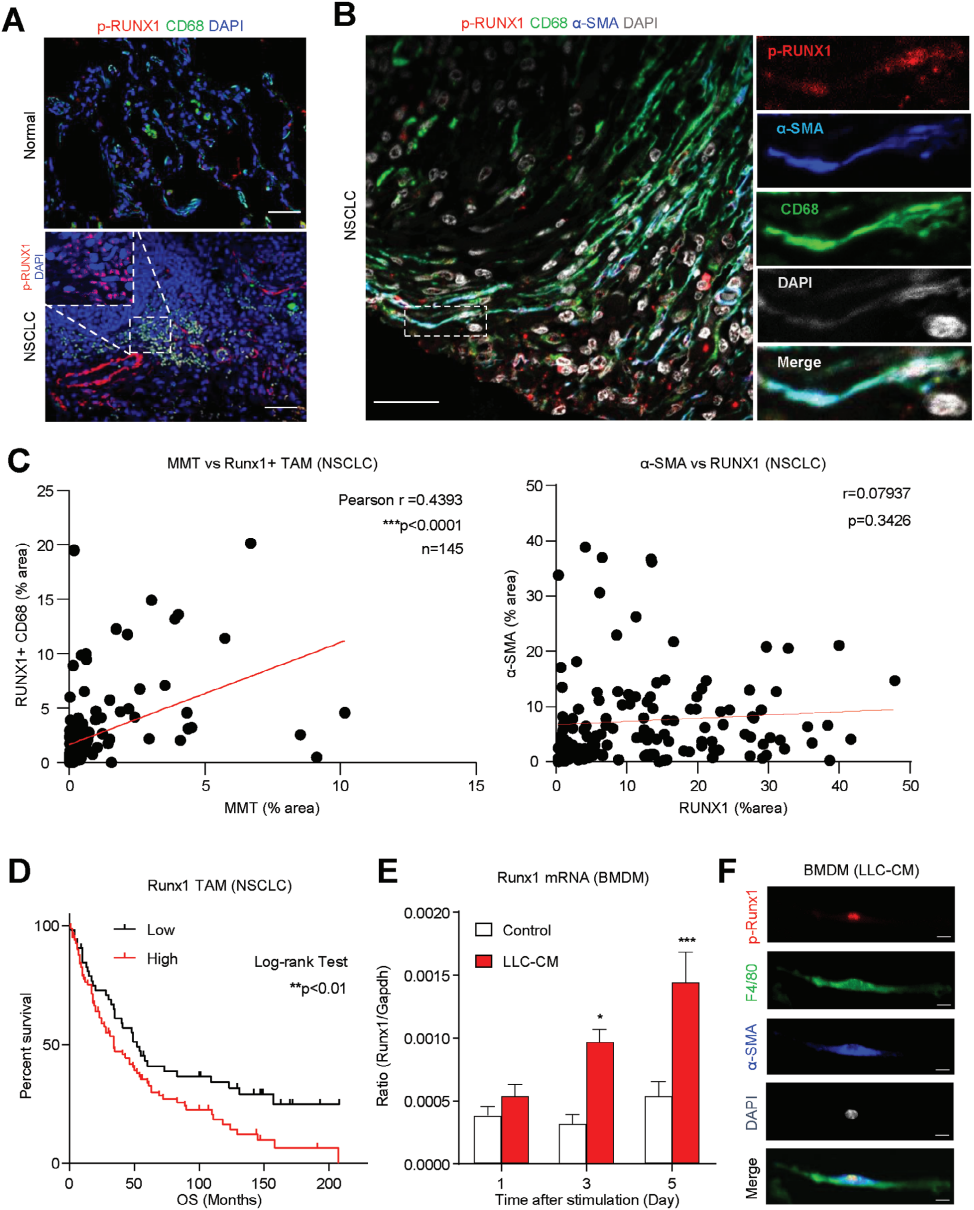
Runx1 expression is associated with MMT development in TME. A) High level of p‐Runx1 expression is observed in human TAMs within lung adenocarcinoma biopsy but is absent in resident macrophages of normal lung tissue. B) Multiplex IHC staining and confocal imaging reveal p‐Runx1+ TAMs undergoing MMT in NSCLC. C) A strong positive correlation is detected between the abundance of Runx1+ TAMs and MMTs in NSCLC but is absent between Runx1 and α‐SMA alone. D) High levels of Runx1+ TAMs are associated with patient mortality in NSCLC (*n* = 145). E,F) Runx1 is progressively induced in macrophages during MMT development under cancer conditions in vitro, indicating a potential role of Runx1 in MMT regulation in NSCLC (*n* = 4; ^***^ *P* < 0.001 vs Day 5 control, ^*^ *P* < 0.05 vs Day 3 control). Scale bar, A,B, 50 µm, F, 25 µm.

By immunofluorescence assay, we found that p‐RUNX1 was highly expressed in the human tumor‐associated macrophages (TAMs) of a lung adenocarcinoma biopsy but absent in the resident macrophages of a normal lung tissue (**Figure** **2**A). Moreover, multiplex Immunohistochemistry (IHC) staining with confocal imagining confirmed the p‐RUNX1^+^ TAMs were undergoing MMT in NSCLC, suggested by their strong expression of CAF marker α‐SMA (Figure 2B). Our cohort study detected a strong positive correlation between the abundance of RUNX1^+^ TAMs and MMTs in NSCLC (***P*** < 0.0001; ***r*** = 0.4393) but absent between the abundance of RUNX1 and α‐SMA alone (Figure 2C), and high level of RUNX1^+^ TAMs was associated with the mortality of NSCLC patients (Figure 2D). Importantly, we detected that p‐Runx1 was progressively induced in the macrophages undergoing cancer condition‐driven MMT on the BMDMs under LLC conditioned medium (LLC‐CM) stimulation in vitro (Figure 2E,F), suggesting a potential role of Runx1 in the regulation of MMT development in NSCLC.

### 2.3 Runx1 is a Novel Smad3 Direct Target in TGF‐β1‐induced MMT

**Figure 3:**
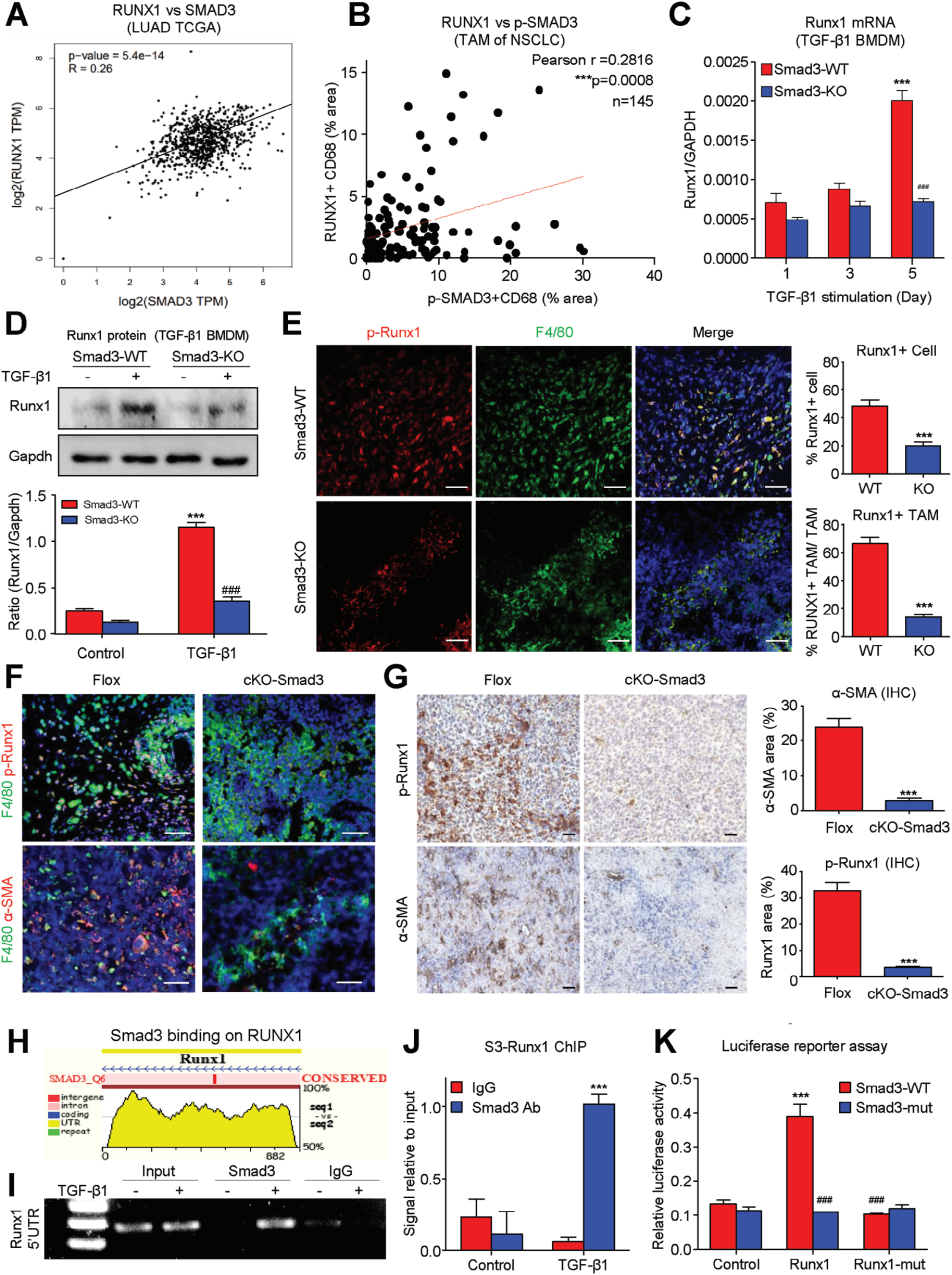
Runx1 as a novel Smad3 direct target in TGF‐β1‐driven MMT. A) A strong positive correlation between RUNX1 and SMAD3 is observed in lung adenocarcinoma TCGA cohort at the transcriptome level. B) The correlation is further confirmed in NSCLC cohort through multiplex IHC staining at the protein level. Runx1 is Smad3‐dependently up‐regulated in TGF‐β1‐stimulated BMDMs in vitro, detected by C) real‐time PCR, D) Western blot analysis, and E) immunofluorescence assay. (3C: *n* = 4; ^***^ *P* < 0.001 vs Day 1 Smad3‐WT, ### *P* < 0.001 vs Day5 Smad3‐WT, 3D: *n* = 4; ^***^ *P* < 0.001 vs Smad3‐WT Control, ### *P* < 0.001 vs TGF‐β1 treated Smad3‐WT) and in TAM of LLC‐tumor in vivo (*n* = 5; ^***^ *P* < 0.001 vs WT). F) Macrophage‐specific Smad3 deletion in Smad3 cKO mice (LysM^Cre^Smad3^fl/fl^) dramatically suppresses p‐Runx1 expression and MMT process of TAMs compared to control (Smad3^flox/flox^) mice, G) contributing to reduced Runx1 expression and CAF formation (*n* = 5; ^***^ *P* < 0.001 vs Control). H) A potential direct binding site for Smad3 protein on the 5′ untranslated region of Runx1 is identified at the genomic level using the ECR browser. I,J) The binding site is experimentally confirmed in BMDMs undergoing TGF‐β1‐driven MMT in vitro (*n* = 4; ^***^ *P* < 0.001 vs Control). K) Deletion of corresponding binding motif (Runx1‐mut: Figure S4B, Supporting Information) or Smad3 phosphorylated sites (Smad3‐mut) effectively halted Runx1 transcription, establishing Runx1 as a novel downstream transcription factor of TGF‐β1/Smad3 signaling for the precise regulation of MMT formation in TME (*n* = 4; ^***^ *P* < 0.001 vs Control, ###*P* < 0.001 vs Smad3‐WT on Runx1). Scale bar, 50 µm.

Since TGF‐β1/Smad3 signaling was reported to be crucial for MMT formation in NSCLC, we examined whether Runx1 is involved in the regulatory pathway. Interestingly, we detected a strong positive correlation between Runx1 and Smad3 in the lung adenocarcinoma TCGA cohort at transcriptome level (***p*** = 5.4 × 10^−14^; **Figure** **3**A), which was further confirmed in our NSCLC cohort with multiplex IHC staining at protein level (***p*** = 0.0008; Figure 3B; Figure S2, Supporting Information). Importantly, we demonstrated that Runx1 was Smad3‐dependently up‐regulated in the TGF‐β1‐stimulated BMDMs in vitro (Figure 3C,D) and MMTs of LLC‐tumor in vivo (Figure 3E). The regulatory role of macrophage Smad3 on Runx1 and associated MMT level was further confirmed on conditional knockout (cKO) mice of Smad3 (LysM^Cre^Smad3^fl/fl^) compared to their control, where locus of X‐over P1 (loxP) sites were introduced to flank exons 2 and 3 for Smad3 (Smad3^flox/flox^).^[^ ^21^ ^]^ Encouragingly, macrophage‐specific Smad3 deletion dramatically suppressed p‐Runx1 expression and MMT of TAM (Figure 3F), leading to the reduction of p‐Runx1 level and CAF formation (Figure 3G; Figure S3, Supporting Information). More importantly, we identified a potential direct binding site of Smad3 protein on the 5′ untranslated region (5′ UTR) of Runx1 at genomic level by ECR browser (Figure 3H), which was experimentally confirmed on the BMDMs undergoing TGF‐β1‐driven MMT in vitro (Figure 3I,J). Notably, this Smad3 binding was unique to the 5′ UTR, with no detectable binding observed elsewhere in the Runx1 gene (Figure S4A, Supporting Information). Further confirmation from a dual reporter assay demonstrating that Smad3‐mediated Runx1 transcription was inhibited either by deletion of the Smad3 binding site (Runx1‐mutant) or by mutation of the Smad3 phosphorylation site (Smad3‐mutant) (Figure 3K; Figure S4B, Supporting Information). Thus, Runx1 represents a novel TGF‐β1/Smad3 direct downstream transcription factor for precise regulation of MMT formation in TME.

### 2.4 Silencing of Macrophage‐Specific TGF‐β1/Smad3/Runx1 Signaling Prevents MMT In Vivo

**Figure 4:**
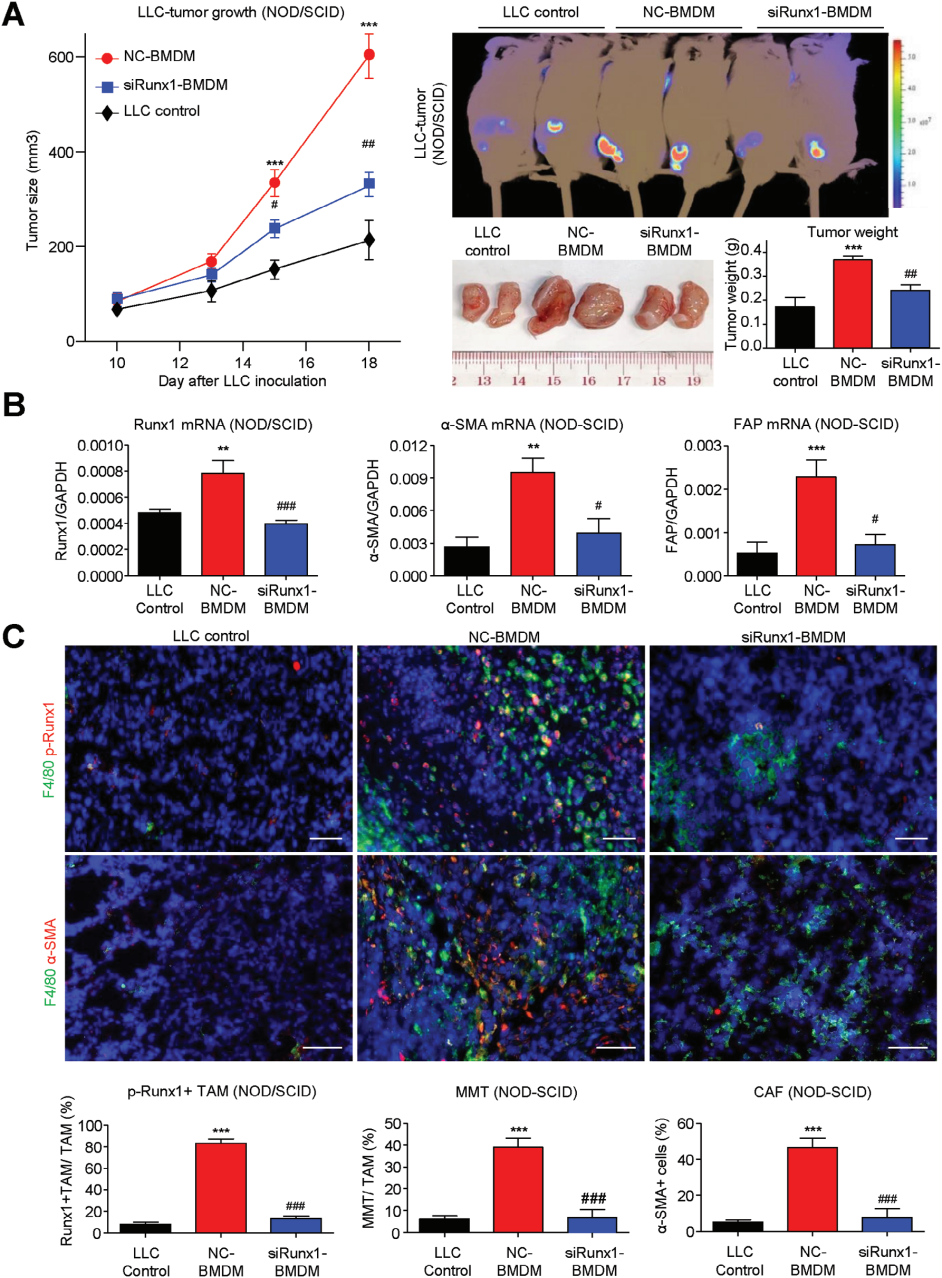
Macrophage‐specific Runx1 is essential for MMT formation in vivo. A–C) Adoptive transfer of Runx1‐silenced BMDMs into NOD/SCID mice with LLC tumors dramatically reduces MMT‐driven accelerated tumor growth, and tumor size was significantly reduced in mice receiving siRunx1‐BMDM compared to NC‐BMDM. B,C) MMT‐driven CAF formation (CAF markers α‐SMA and FAP) are downregulated in the siRunx1‐BMDM group compared to the NC‐BMDM group, showing that targeting Runx1 in BMDMs effectively blocks TGF‐β1‐induced MMT and CAF marker expression in vivo (*n* = 5; ^**^ *P* < 0.01 vs LLC Control, ^***^ *P* < 0.001 vs LLC Control, #*P* < 0.05 vs NC‐BMDM, ##*P* < 0.01 vs NC‐BMDM, ###*P* < 0.001 vs NC‐BMDM). Scale bar, 50 µm.

To explore the function of Runx1 in MMT, we adoptively transferred BMDMs with siRNA‐mediated Runx1 knockdown (siRunx1‐BMDM, Figure S5, Supporting Information) or nonsense‐treated control (NC‐BMDM) or saline only (LLC control) together with LLC lung cancer cells (1:1 ratio) into macrophage malfunction nonobese diabetic/severe combined immunodeficiency (NOD/SCID) mice. Encouragingly, we found that the accelerated tumor growth (**Figure** **4**A), Runx1 expression, MMT and CAF formation (α‐SMA, FAP Figure 4B,C) in the NC‐BMDM group were significantly suppressed in the mice that received Runx1‐silenced BMDMs (siRunx1‐BMDM), revealing the essentialness of macrophage‐specific Runx1 in MMT development.

### 2.5 Runx1 Promotes CAF Signatures at Transcriptional Level During MMT

**Figure 5:**
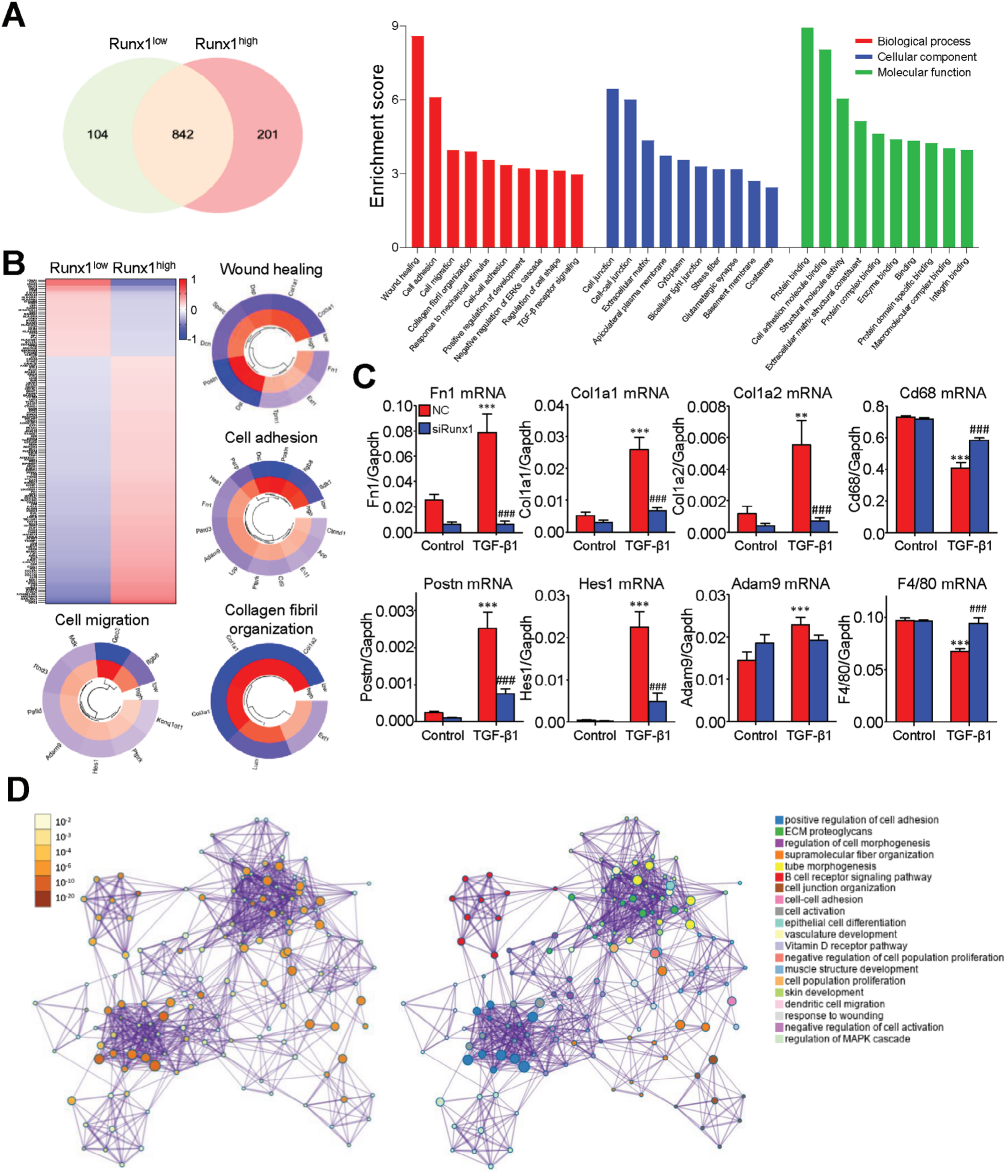
Runx1 promotes CAF signatures at transcriptional level during MMT. A) There were 201 DEGs specifically upregulated in the human Runx1^high^ TAMs of NSCLC scRNA‐seq dataset. B) GO analysis highlighted strong links between DEGs specific to the Runx1^high^ TAMs and CAF functionalities, spotlighting CAF markers such as Fn1, Col1a1, Col1a2, Postn, Hes1, and Adam9 involved in processes like wound healing, cell adhesion, migration, and collagen organization. C) Silencing Runx1 successfully reversed expression levels of CAF and macrophage markers in the TGF‐β1‐treated BMDMs, demonstrating their role as downstream effectors of Runx1 during MMT development (*n* = 4, ^**^ *P* < 0.01 vs NC‐Control, ^***^ *P* < 0.001 vs NC‐Control, ###*P* < 0.001 vs NC‐TGF‐β1). D) Unbiased Metascape transcriptome analysis confirmed CAF‐related functions including ECM proteoglycans, tube morphogenesis, supramolecular fiber organization, cell adhesion, and activation are the major functions of Runx1^high^ TAMs as shown in two clusters with the most connection, uncovering apathogenic role of Runx1 in the transition of TAMs into pro‐tumoral CAFs via MMT.

To identify the functional role and clinical relevance of Runx1 in MMT, we compared the transcriptomic profiles of Runx1^high^ and Runx1^low^ TAMs in the human NSCLC datasets with single‐cell resolution. Interestingly, we found 201 differentially expressed genes (DEGs) were specifically upregulated in the Runx1^high^ TAMs (**Figure** **5**A) which were highly associated with signatures and functions of CAF, including wound healing, cell adhesion, migration, and collagen fibril organization showing by GO analysis (Figure 5B). These CAF‐signatures (Fn1, Collagen, type I, alpha 1 (Col1a1), alpha 2 (Col1a2), Periostin (Postn), hairy and enhancer of split‐1 (Hes1), but not Disintegrin and metalloproteinase domain‐containing protein 9 (Adam9)) are downstream factors of Runx1, as demonstrated by experimental silencing of Runx1 in TGF‐β1 treated BMDMs (Figure 5C). Further Metascape analysis was performed to elucidate the major functions of Runx1^high^ TAMs as categorized into clusters. Surprisingly, the two major clusters formed are related to CAF functions, i.e., Extracellular matrix (ECM) proteoglycans, tube morphogenesis, and supramolecular fiber organization in one cluster, while cell adhesion and activation formed another cluster (Figure 5D).

**Figure 6:**
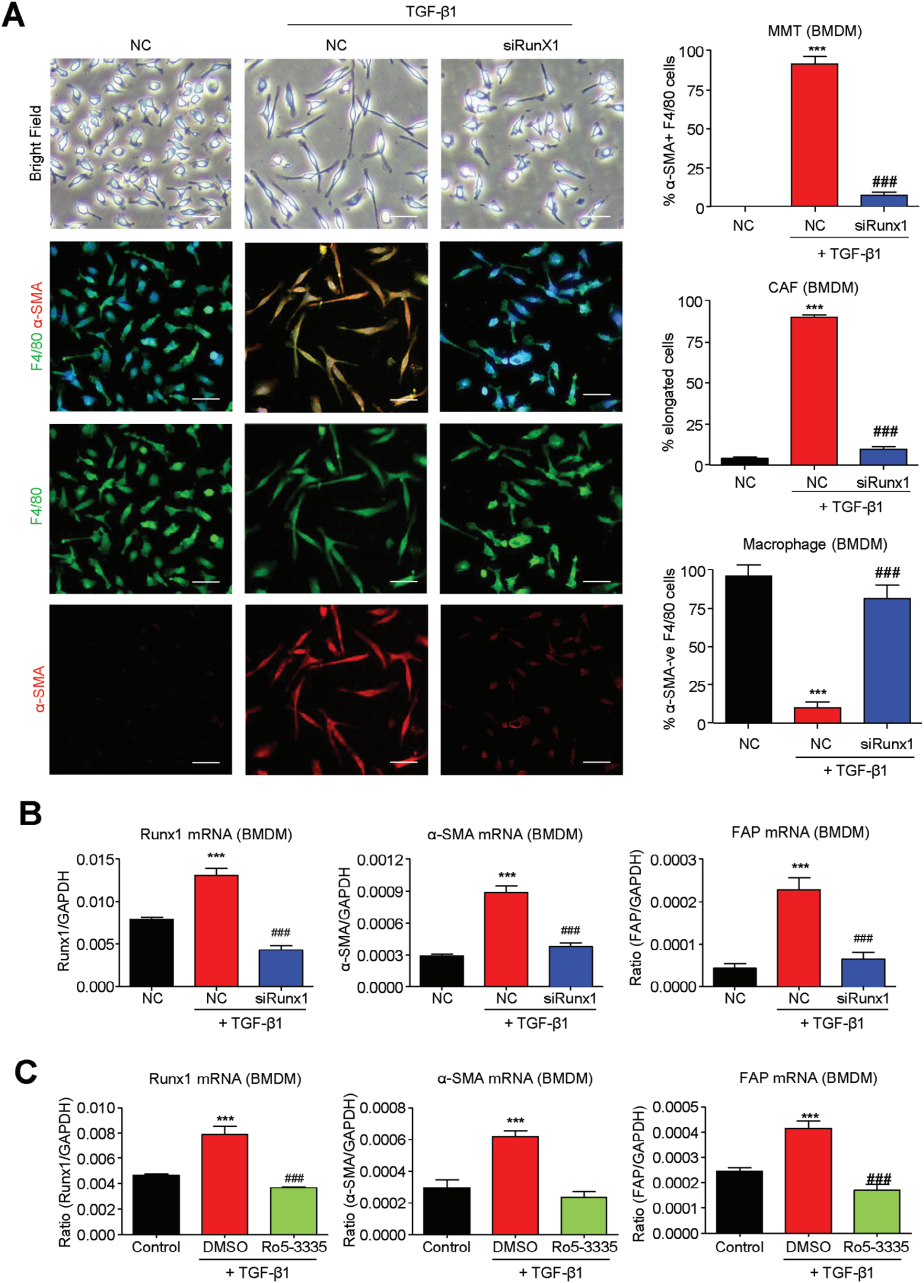
Runx1 inhibition effectively blocks TGF‐β1‐induced MMT in vitro. A,B) CAF‐like morphology and CAF markers (α‐SMA, FAP) induced by 5‐day TGF‐β1 stimulation in BMDMs are largely suppressed by macrophage specific silencing of Runx1 (*n* = 4, ^***^ *P* < 0.001 vs NC‐Control, ###*P* < 0.001 vs NC‐TGF‐β1). C) Importantly, the MMT suppression on BMDMs can be reproduced by pharmaceutical Runx1 inhibition using a Runx1 specific inhibitor 5 µM Ro5‐3335 (*n* = 4, ^***^ *P* < 0.001 vs Control, ###*P* < 0.001 vs DMSO‐TGF‐β1). Scale bar, 50 µm.

Therefore, we examined the therapeutic potential of macrophage specific Runx1 inhibition for MMT prevention in vitro. Encouragingly, we demonstrated that both genetic silencing (**Figure** **6**A,B) and pharmaceutical inhibition with Runx1 specific inhibitor Ro5‐3335 (Figure 6C) on BMDMs effectively blocked TGF‐β1‐driven MMT formation in vitro, showing by the reduction of CAF morphology and marker expression (α‐SMA, FAP) in the 5‐day TGF‐β1‐stimulated BMDMs, suggesting Runx1 specific inhibitor can be further developed as a novel MMT targeted therapy for lung cancer.

### 2.6 Development of a Runx1‐Based MMT‐Targeted Therapy for Lung Cancer

**Figure 7:**
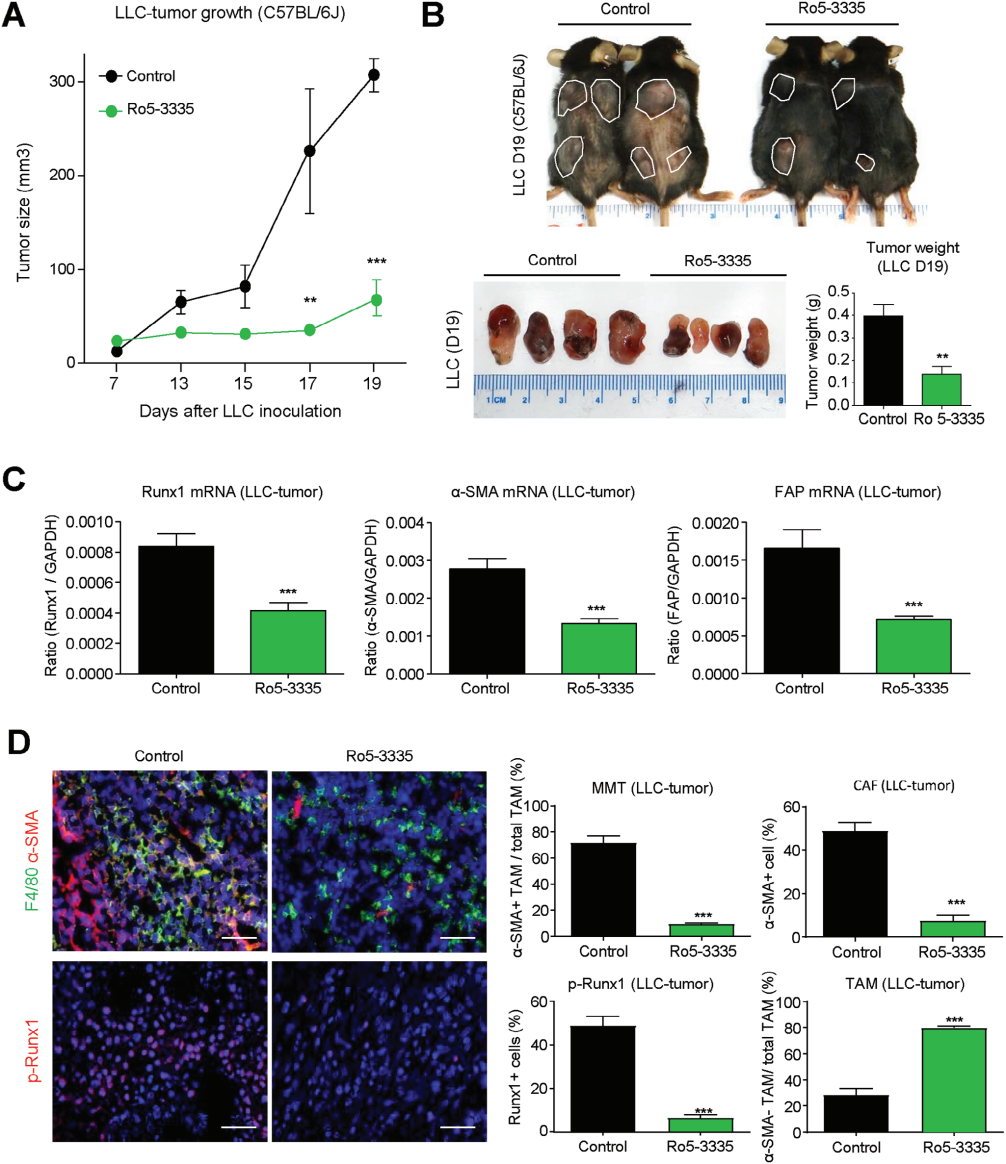
Therapeutic efficiency of Runx1‐based MMT‐targeted therapy for lung cancer. A,B) Runx1 inhibitor Ro5‐3335 (5 mg kg^‐1^/day) effectively blocks LLC tumor progression. C) Tumoral Runx1 and CAF markers (α‐SMA and FAP) expression were largely suppressed by Ro5‐3335 treatment. D) Runx1 inhibitor reduces CAF formation, MMT, and p‐Runx1 protein expression in LLC tumors, as evidenced by the significant decrease of CAF (α‐SMA+), MMTs (α‐SMA+ F4/80+), and p‐Runx1+ cells in the Ro5‐3335 treated group compared to the PBS control group, demonstrating the therapeutic potential ofRunx1 targeted therapy forblocking MMT‐driven cancer development in clinical NSCLC. (*n* = 5, ^**^ *P* < 0.01 vs Control, ^***^ *P* < 0.001 vs Control) Scale bar, 50 µm.

We evaluated the therapeutic potential of Runx1‐based MMT targeted therapy on lung cancer by daily injection of Runx1 specific inhibitor Ro5‐3335 (5 mg kg^−1^ i.p.) into immunocompetent mice bearing syngeneic lung cancer LLC. We observed that treatment with Ro5‐3335 effectively blocked LLC tumor progression (**Figure** **7**A,B) via inhibiting CAF formation (α‐SMA and FAP) in vivo (Figure 7C), without any observed adverse effects (Figure S6, Supporting Information). Importantly, by multicolor immunostaining, we confirmed that pharmaceutical systemic inhibition of Runx1 specifically blocked MMT‐driven CAF formation in the lung cancer TME, shown by a dramatic reduction of MMTs (α‐SMA^+^ F4/80^+^) in the Ro5‐3335 treated group in vivo (Figure 7D). To note, the antitumor effect of systemic inhibition of Runx1 was better than the macrophage specific strategy in vivo, implying additional Runx1‐dependent mechanisms beyond MMT contributing to the cancer progression which can be further investigated. In overall, our results uncovered a new pathogenic role of Runx1 in MMT development, representing a novel druggable target for the development of targeted therapy against the MMT‐driven formation of tumor‐promoting CAFs in clinical NSCLC.

## 3 Discussion

In our previous studies, we reported that TGF‐β/Smad3 signaling is a key regulator of the pro‐tumoral microenvironment^[^ ^10^, ^11^, ^22^ ^]^ and essential for initiating macrophage‐myofibroblast transition under chronic inflammatory diseases, including cancer.^[^ ^14^, ^18^, ^23^, ^24^ ^]^ However, targeting Smad3 would also suppress T‐cell anticancer immunity.^[^ ^25^ ^]^ Therefore, we aimed to search for macrophage‐specific TGF‐β/Smad3 signaling by identifying and characterizing its downstream pathogenic mediators as potential therapeutic targets that could specifically block MMT while preserving the anticancer activities of Smad3. Encouragingly, we have identified numerous cell type‐specific TGF‐β/Smad3 signaling pathways as novel and effective therapeutic targets for fibrotic diseases.^[^ ^10^, ^14^, ^15^, ^18^, ^24^, ^26^, ^27^ ^]^ Therefore, we would extensively investigate the yet unexplored macrophage‐specific TGF‐β/Smad3 signaling pathways in lung cancer to identify potential therapeutic targets for precise inhibition of MMT formation in TME.

By analyzing the transcriptome dynamics of TAM at single‐cell resolution, we identified Runx1, a transcription factor associated with hematopoiesis,^[^ ^28^ ^]^ as a key driver for MMT under LLC‐tumor microenvironment.^[^ ^22^ ^]^ Interestingly, although there have been indications of a connection between Smad3 and Runx1,^[^ ^29^, ^30^ ^]^ their direct molecular mechanism remains unexplored. Remarkably, through an MMT specific Chromatin immunoprecipitation Polymerase Chain Reaction (ChIP‐PCR) assay, we identified direct binding of Smad3 to the 5′UTR of Runx1, which confirmed that Runx1 is a direct target of Smad3, thereby revealing the presence of a macrophage‐specific TGF‐β/Smad3/Runx1 signaling pathway. The essentialness of macrophage specific TGF‐β/Smad3/Runx1 signaling pathway for initiating MMT is validated in vivo, with our newly generated macrophage specific condition Smad3‐KO mice (LysM^Cre^Smad3^fl/fl^), where macrophage specific Smad3 deletion effectively prevents Runx1 induction and MMT driven CAF formation in LLC tumor. Importantly, both pharmaceutical inhibition and genetic silencing of Runx1 effectively blocked MMT in LLC tumors in vivo and TGF‐β1‐stimulated BMDMs in vitro, clearly demonstrating the therapeutic potential of targeting macrophage specific TGF‐β/Smad3/Runx1 signaling to suppress MMT driven tumor growth.

Although Runx1 is reported as a transcription factor involved in microglia differentiation^[^ ^31^ ^]^ and stem cell lineage commitment,^[^ ^32^ ^]^ its role in CAF development remains largely unknown. Interestingly, recent studies have revealed a potential link between Runx1 and CAF differentiation, where Runx1‐dependent stromal gene signature was identified in CAFs isolated from 4T1 tumors compared to normal fibroblasts in vivo,^[^ ^33^ ^]^ and Runx1 silencing blocked TGF‐β1‐induced differentiation of human lung fibroblasts into myofibroblasts in vitro.^[^ ^34^ ^]^ In line with this notion, we observed a dramatic increase in Runx1 expression in NSCLC and MMTs at the single‐cell resolution and found its positive correlation with CAF marker expression in TCGA cohort of lung adenocarcinoma. This study provides the first evidence of the regulatory role of Runx1 in MMT‐driven CAF formation and emphasizes its potential as a therapeutic target for MMT‐driven tumor growth. In our in‐house NSCLC cohort, we confirmed Runx1 expression on human MMTs, and its TAM specific expression is positively correlated with the abundance of MMTs at the protein level, which is further supported by the Runx1 expression detected in LLC‐CM stimulated BMDMs in vitro. These findings suggest that Runx1 is not only involved in hematopoiesis but also plays a critical role in the MMT process, which contributes to CAF formation and tumor progression. Importantly, we have shown that inhibition of Runx1 effectively blocked MMT in LLC tumors in vivo and TGF‐β1‐stimulated BMDMs in vitro, which significantly contributed to the suppression of tumor growth in LLC‐bearing mice. This indicates that targeting Runx1 could be a potential strategy to specifically block MMT‐driven CAF formation while preserving the anticancer activities of Smad3.

In fact, an interesting phenomenon was observed in our in vivo study, where Ro5‐3335 mediated Runx1 inhibition led to a significant decrease in Runx1 expression at the protein level in the treated mice. To note, Ro5‐3335 was developed to prevent the physical interaction between Runx1 and its co‐factor Core Binding Factor β (CBFβ), thereby inhibiting Runx1‐mediated regulatory actions on its downstream factors in the nucleus at the transcriptional level.^[^ ^35^ ^]^ Other study also reported that Runx1 can regulate its expression via triggering demethylation of the Runx1 promoter.^[^ ^36^ ^]^ Therefore, targeting Runx1 activity at protein level would lead to its repression at transcription level. Moreover, we observed a broader anti‐tumor effect of R05‐3335 compared to the siRNA‐mediated macrophage specific Runx1 inhibition in vivo. It is because systematic Ro5‐3335 treatment can target all Runx1 expressing cells in the TME, whereas our siRNA assay was only restricted to the adoptively transferred macrophages. Previous studies have underscored the pro‐tumoral role of Runx1 not only in macrophages but also in various cell types, including cancer cells.^[^ ^37^, ^38^ ^]^ Thus, Ro5‐3335 provides a global inhibition of Runx1 and manifests a more profound anti‐tumor effect compared to macrophage specific inhibition of siRNA. Indeed, the siRNA was only transfected into BMDM once before adoptive transfer, offers a transient effect in contrast to the sustained impact of daily‐applied Ro5‐3335. Given this limitation, we recommend employing CRISPR‐Knockout technology in future studies to ensure longer‐lasting silencing effects for fair comparison. Another aspect to consider is the role of Runx1 in tumor angiogenesis.^[^ ^39^ ^]^ Given that angiogenesis is crucial for tumor growth and metastasis by providing nutrients and oxygen to tumor cells and is notably amplified by the adoptive transfer of BMDM‐derived MMTs into LLC tumors,^[^ ^22^ ^]^ potential involvement of Runx1 in this process could further intensify its influence on tumor progression.

Importantly, this study successfully identified Runx1 as a better therapeutic target from the macrophage specific Smad3 downstream signaling for development of MMT precision medicine. Indeed, the potential side‐effects of Smad3 inhibitor on cancer patients due to the impairment of host T cell immunity during systemic Smad3 inhibition,^[^ ^12^, ^25^, ^40^ ^]^ which largely limits its further translation. In this study, we showed that Runx1 inhibitor, Ro5‐3335, induced no side effect on the cancer‐bearing mice. It is consistent to another study where the cancer‐free mice were received a much higher dose (300 mg kg^−1^/d) for a month.^[^ ^35^ ^]^ Although Runx1 is suggested to be an important regulator for hematopoiesis, a new study demonstrated that its hematopoietic function can be replaced by other Runx1‐independent mechanisms (e.g., GATA2) in the Runx1‐KO hematopoietic stem cells.^[^ ^41^ ^]^ In addition, our in vitro experiments further demonstrated that Runx1 inhibition can effectively block MMT without cytotoxicity on the primary macrophages. Moreover, Runx1 also has been implicated in various malignancies associated with CAF abundance, including breast, bladder, cervical, and rectal cancer,^[^ ^33^, ^42^ ^]^ suggesting its potential involvement in other cancer types by accelerating the pathogenic MMT process for CAF formation. Investigating the role of Runx1 in different malignancies and its association with MMT and other signaling pathways could provide valuable insights for the development of targeted therapies. Therefore, a comprehensive understanding of the role of Runx1 in various aspects of tumor biology, including MMT‐driven CAF formation, angiogenesis, and immune cell regulation, is essential for the development of effective therapeutic strategies targeting Runx1.

Moreover, α‐SMA+ fibroblasts are highly heterogenous including CAFs originated from different sources beyond the MMT, therefore both pro‐tumor and anti‐cancer subpopulations are reported. α‐SMA+ fibroblasts were found to show tumor‐restraining activity in solid cancer showing by α‐SMA+ cells depletion assay in mice.^[^ ^43^ ^]^ Fascinatingly, an in‐depth scRNA‐seq‐based analysis revealed that an IL6‐releasing α‐SMA+ CAF subset is associated with the gemcitabine resistance in pancreatic cancer.^[^ ^44^ ^]^ In addition, new studies demonstrated that α‐SMA+ CAFs linked to lymph node metastases^[^ ^45^, ^46^ ^]^ and a poorer outcome^[^ ^47^ ^]^ in breast cancer. Recently, scRNA‐seq analysis identified a FAP+ α‐SMA+ CAF subset for promoting the TGF‐β1‐driven resistance in immunotherapy.^[^ ^48^ ^]^ MMT might also play a role in other cancer types, which is yet to be identified and worth for follow‐up studies. In addition, from the experience in kidney diseases, macrophages can regulate myofibroblast formation via both direct and indirect pathways.^[^ ^49^, ^50^ ^]^ Additional research has indicated that tumor‐associated macrophages are capable of inciting myofibroblast activation in the tumor microenvironment through their secretome.^[^ ^51^, ^52^, ^53^ ^]^ The pronounced reduction of total α‐SMA seen in LLC‐bearing macrophage‐specific Smad3 conditional knock‐out mice intimates a potential function of Smad3 in the macrophage‐driven activation of CAF in NSCLC. This implication warrants further investigation in future.

In conclusion, our study discovered a novel macrophage‐specific TGF‐β1/Smad3/Runx1 signaling which is essential for promoting MMT in TME, serving as a precise therapeutic target of protumoral CAF for anticancer therapy. Further research is needed to elucidate the safety and therapeutic efficiency of Runx1‐based MMT‐targeted therapy as well as its potential synergy in combination with conventional therapies for NSCLC.

## 4 Experimental Section

### NSCLC Cohort

Formalin‐fixed and paraffin‐embedded (FFPE) tissue microarray (TMA) and frozen sections of primary NSCLC were collected from patients who underwent lobectomy at Prince of Wales Hospital, affiliated with The Chinese University of Hong Kong (CUHK). The study was conducted in accordance with approved protocols (Reference No. 2019.368) by the Clinical Research Ethics Committee of Joint Chinese University of Hong Kong‐New Territories East Cluster (CREC CUHK‐NTEC) and adhered to the principles outlined in the Declaration of Helsinki. Written informed consent was obtained from all participating patients.

### Immunohistochemistry and Opal Multiplexing

To analyze the expression level of Runx1 and the abundance of CAFs in tumoral tissues, immunohistochemistry was conducted on 5 µm FFPE sections of human NSCLC and experimental LLC tumors. A microwave‐based antigen retrieval method was employed to expose and unmask the target antigens in the tissue sections. The samples were then incubated with primary antibodies against Runx1 (sc‐365644, Santa Cruz) or α‐smooth muscle actin (α‐SMA, A5691; Sigma). Subsequently, a secondary antibody conjugated to horseradish peroxidase (HRP, Dako) was added, producing a visible signal with the substrate 3,3′‐diaminobenzidine (DAB, Fujifilm). The results were visualized using a Nikon Ni‐U light microscope.

For Opal multiplexing, samples were incubated overnight at 4 °C with primary antibodies, followed by the development of fluorescence using the OPAL 4‐color IHC kit (Perkin‐Elmer) according to the manufacturer's instructions. Images were acquired from four to six distinct areas of each sample using the Mantra quantitative pathology workstation (Perkin–Elmer) and analyzed with the inForm image analysis software (Perkin–Elmer). The area stained for MMTs (CD68+ α‐SMA+ cells) and Runx1 in the TMA was normalized based on the total tissue area in each image. 3D imaging was performed using a confocal microscope (Carl Zeiss LSM 880).

### Transcriptomic Analysis

The human NSCLC dataset in cloupe format was obtained from the 10X Genomics website (https://support.10xgenomics.com/single‐cell‐gene‐expression/datasets). Runx1^high^ and Runx1^low^ TAMs were identified by gating CD68+ cells with Runx1 expression using a cutoff value of > 3.0, employing the Loupe Cell Browser 6.0.0 version. For correlation analysis of gene expression in The Cancer Genome Atlas‐lung adenocarcinoma (TCGA‐LUAD) dataset, the web‐based analysis tool GEPIA (Gene Expression Profiling Interactive Analysis) was utilized.^[^ ^42^ ^]^ Upregulated DEGs of Runx1^high^ were submitted to Database for Annotation, Visualization, and Integrated Discovery Bioinformatics resources (DAVID v6.8) for Gene Ontology biological processes enrichment analysis. The relationships between the GO terms were further captured by Metascape Analysis,^[^ ^54^ ^]^ a subset of enriched terms has been selected and rendered as a network plot according to the default setting, where terms with a similarity > 0.3 were connected by edges. The terms with the best *p*‐values from each of the 20 clusters, with the constraint that there are no more than 15 terms per cluster and no more than 250 terms in total were used. The network was visualized using Cytoscape5.

### Immunofluorescence

Immunostaining was performed on cultured BMDMs and frozen sections of human and mouse tissues fixed by 2% paraformaldehyde following a previously described protocol.^[^ ^18^, ^22^ ^]^ In brief, sections were labeled overnight with various combinations of directly conjugated primary antibodies as follows: fluorescein isothiocyanate (FITC)‐conjugated anti‐CD68 antibody (sc‐20060 FITC, Santa‐cruz), FITC‐conjugated anti‐F4/80 (11‐4801‐81, eBioscience), Cy3‐conjugated α‐SMA antibody (C6198, Sigma), Runx1 (sc‐365644, Santa Cruz), p‐Runx1(Ser397) (PA5‐105609, Invitrogen) detected with APC (A21240, Invitrogen) or PE‐conjugated secondary antibodies (A11003, Invitrogen). Sections were washed and, in some cases, DNA was counterstained with DAPI and observed under a fluorescence microscope (Carl Zeiss Axio Observer Z1) or confocal microscope (Carl Zeiss LSM 880).

### Experimental Animals

C57BL/6J, NOD/SCID (8‐ to 10‐week‐old), Smad3‐deficient (Smad3^−/−^),^[^ ^25^ ^]^ macrophage‐specific Smad3‐deleted mice (LysM^Cre^Smad3^fl/fl^) were used. C57BL/6 mice bearing homozygous loxP‐flanked Smad3 (Smad3^flox/flox^) alleles (generated by Cyagen Biosciences, CA, US) were crossed to mice with lysozyme M promoter‐driven Cre (lysM‐cre) (JAX stock number: 004781) to obtain macrophage‐specific Smad3‐deleted mice (LysM^Cre^Smad3^fl/fl^). Mice were bred or purchased from The Chinese University of Hong Kong Laboratory Animal Services Centre. All experimental procedures were approved by the Animal Ethics Experimental Committee of The Chinese University of Hong Kong (AEEC Ref No.: 20‐019‐GRF) and conducted following the guidelines of AEEC and local regulations.

*Treatment with Ro5‐3335*: Mice with Smad3+/+ genotype bearing LLC‐tumors (administered subcutaneously) were randomly separated into two sets (*n* = 5). One group was given Ro5‐3335 (#4694, Tocris Bioscience) at a dosage of 5 mg kg^‐1^ via intraperitoneal injection (i.p.) on a daily basis until the tumor was harvested. The control group, on the other hand, was treated with a solvent control comprising 0.05% dimethylsulfoxide.

*Adoptive Transfer Studies*: In order to determine Runx1's specific function, Smad3+/+ BMDM were introduced to both scramble and siRunx1 siRNAs, producing NC‐ and siRunx1‐BMDM for the transfer study, as described in references.^[^ ^18^, ^22^, ^55^ ^]^ PBS, NC‐, or siRunx1‐BMDM were combined with LLC cells at an equal ratio (2 × 10^6^ cells per mouse). These were then subcutaneously injected into NOD–SCID mice with impaired macrophage function, creating three distinct groups: LLC control, NC‐BMDM, and siRunx1‐BMDM (*n* = 5). Every two days, tumor sizes were assessed using a Vernier caliper and the volume was determined as: Volume (mm^3^) = 0.5 (length x width squared). Prior to tumor extraction, the bioluminescence of LLC‐luc tumors was analyzed using the IVIS Spectrum system (by Caliper, Xenogen).

### Cell Culture and Treatment Procedures

LLC cells were grown in complete Dulbecco's Modified Eagle Medium/Nutrient Mixture F‐12 (DMEM/F12) containing 10% fetal bovine serum, 100 U mL^−1^ penicillin, and 100 µg mL^−1^ streptomycin. Bone marrow‐derived macrophages (BMDMs) were isolated from the tibia, femur, and ilium bones according to established protocols.^[^ ^14^, ^22^, ^24^ ^]^ Briefly, bone marrow cells were differentiated for 7 days in DMEM supplemented with 10% heat‐inactivated FBS, 100 U mL^−1^ penicillin, 100 µg mL^−1^ streptomycin, and 50 ng mL^−1^ recombinant mouse macrophage colony‐stimulating factor (Gibco). BMDMs were exposed to TGF‐β1, LLC‐CM, or Runx1 inhibitors Ro5‐3335 (Tocris Bioscience) to investigate Runx1's role in MMT development. The LLC‐CM was obtained by incubating LLC cells overnight in serum‐free DMEM/F12, followed by filtration using a 0.2 µm nylon membrane.

### Chromatin Immunoprecipitation (ChIP) Assay

BMDMs were exposed to 5 ng mL^−1^ of TGF‐β1 for 2 h, followed by a ChIP assay using the SimpleChIP Enzymatic Chromatin IP Kit (Magnetic Beads) (Cell Signaling, No. 9003), as per the manufacturer's guidelines and as previously reported.^[^ ^22^ ^]^ Briefly, immunoprecipitation was carried out using an anti‐Smad3 antibody (1:100 dilution) and an isotype‐matched IgG antibody as a negative control (Cell Signaling, No. 9523 and No. 3900). The precipitated DNA fragments were detected by PCR with gel electrophoresis, employing specific primers targeting the predicted Smad3 binding site within the conserved region of the mouse Runx1 gene on the 5' UTR: forward primer 5′‐AAACTCGATGACACTCAGGGTA and reverse primer 5′‐CTCGCTCGTGGGTATTTGTG.

### Dual‐Luciferase Reporter Analysis

Using pcDNA3.1+ plasmids, the analysis incorporated both the full‐length sequence of Smad3 (pcDNA3.1‐Smad3‐wildtype) and its altered variant (pcDNA3.1‐Smad3‐mutant). The reporter plasmid psi‐check2, constructed with the Runx1 5′ UTR sequence by Igebio (Guangzhou, China),^[^ ^10^, ^14^ ^]^ included the primary sequence (Runx1) and a modified one where the anticipated Smad3 binding site was removed (Runx1‐mut), as shown in Figure S4B (Supporting Information). Alongside the Renilla expression sequence embedded in psi‐check2 serving as an internal control, these blueprints were co‐introduced into 293T cells utilizing Lipofectamine 2000. After this process, the luciferase outputs were assessed 48 h post‐introduction. The reported activity was determined by comparing firefly to Renilla luciferase outputs. This luciferase reporter analysis was conducted by Landbiology (Guangzhou, China)utilizing the Promega Dual‐Luciferase Reporter System, as in our previous studies.^[^ ^10^, ^14^ ^]^ Data was presented as the average ± SEM multiplier of luciferase over three separate tests.

### Quantitative Real‐Time PCR

RNA extraction, complementary DNA (cDNA) synthesis, and real‐time polymerase chain reaction (PCR) were performed using TRI reagent (Molecular Research Center), a reverse transcription system (Promega), and SYBR Green Supermix (Bio‐Rad), respectively, following the manufacturer's guidelines.^[^ ^14^, ^22^ ^]^ The primers sequences utilized in this study were listed in Table S2 (Supporting Information). The gene expression levels from three independent experiments were normalized to GAPDH and reported as the mean ± standard error of the mean (SEM).

### Statistical Analysis

The data are expressed as means ± standard error of the mean (SEM). Group comparisons were conducted using either the Student's *t*‐test or one‐way analysis of variance (ANOVA), followed by Tukey's post hoc test, depending on the context. Survival analysis was carried out using the log‐rank test, and a *p*‐value below 0.05 was deemed statistically significant. All statistical analyses were performed using the Prism software (Prism 5.0, GraphPad Software, San Diego, CA).

## Conflict of Interest

The authors declare no conflict of interests.

## Supporting information

Supporting Information

## Data Availability

The data that support the findings of this study are available from the corresponding author upon reasonable request.
