# Supplementary material for: Hematopoietic Transcription Factor RUNX1 is Essential for Promoting Macrophage–Myofibroblast Transition in Non‐Small‐Cell Lung Carcinoma
Source: Adv Sci (Weinh). 2023 Nov 15;11(1):2302203. doi: 10.1002/advs.202302203 (PMC10767400; doi:10.1002/advs.202302203)
Supplement: Supplementary file 1 — Supporting Information [file ADVS-11-2302203-s001.pdf]

ADVANCED SCIENCE
Open Access

Supporting Information

for *Adv. Sci.*, DOI 10.1002/advs.202302203

Hematopoietic Transcription Factor RUNX1 is Essential for Promoting Macrophage–Myofibroblast Transition in Non-Small-Cell Lung Carcinoma

*Philip Chiu-Tsun Tang, Max Kam-Kwan Chan, Jeff Yat-Fai Chung, Alex Siu-Wing Chan, Dongmei Zhang, Chunjie Li, Kam-Tong Leung, Calvin Sze-Hang Ng, Yi Wu, Ka-Fai To, Hui-Yao Lan and Patrick Ming-Kuen Tang**

Hematopoietic Transcription Factor RUNX1 is Essential for Promoting Macrophage–Myofibroblast Transition in non-small-cell Lung Carcinoma

Philip Chiu-Tsun Tang[1*], Max Kam-Kwan Chan[1*], Jeff Yat-Fai Chung[1], Alex Siu-Wing Chan[2], Dongmei Zhang[3], Chunjie Li[4], Kam-Tong Leung[5], Calvin Sze-Hang Ng[6], Yi Wu[7], Ka-Fai To[1], Hui-Yao Lan[8], Patrick Ming-Kuen Tang[1]

[1] Department of Anatomical and Cellular Pathology, State Key Laboratory of Translational Oncology, The Chinese University of Hong Kong, Hong Kong

[2] Department of Applied Social Sciences, The Hong Kong Polytechnic University, Hong Kong

[3] College of Pharmacy, Jinan University, Guangzhou, China

[4] Department of Head and Neck Oncology, West China Hospital of Stomatology, Sichuan University, Chengdu, Sichuan, China

[5] Department of Paediatrics, The Chinese University of Hong Kong, Shatin, Hong Kong

[6] Department of Surgery, The Chinese University of Hong Kong, Hong Kong

[7] MOE Key Laboratory of Environment and Genes Related to Diseases, School of Basic Medical Sciences, Xi'an Jiaotong University, Xi'an, China

[8] Department of Medicine and Therapeutics, Li Ka Shing Institute of Health Sciences, The Chinese University of Hong Kong

[*] These authors contributed equally to this work.

Correspondence: Dr. Patrick Ming-Kuen Tang, Department of Anatomical and Cellular Pathology, State Key Laboratory of Translational Oncology, The Chinese University of Hong Kong. Tel: 852-35052349; E-mail: patrick.tang@cuhk.edu.hk

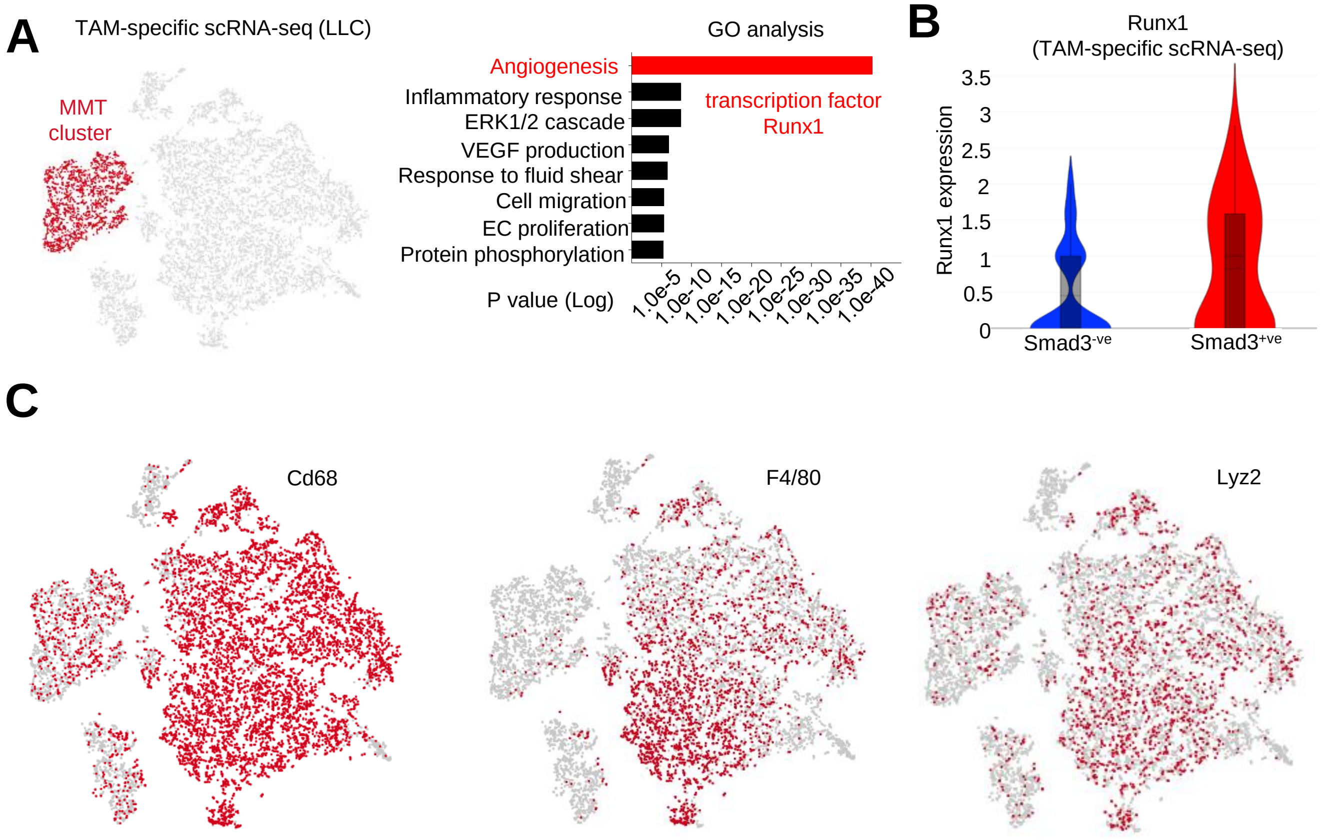

Supplementary Figure S1. Runx1 is the major transcription factor that highly expressed in TAMs undergoing MMT *in vivo*. **(A)** GO analysis revealed that Runx1 is a key transcription factor that highly expressed in the MMT cluster (Red) in our published 10X scRNA-seq dataset *in vivo* [Tang, et al., Adv Sci (Weinh), 2022]. **(B)** Runx1 is markedly up-regulated in the Smad3 positive TAMs in the LLC-tumour *in vivo*. **(C)** Expression level of macrophage lineage markers (Cd68, F4/80, Lyz2) in the macrophage-specific 10X scRNA-seq dataset.

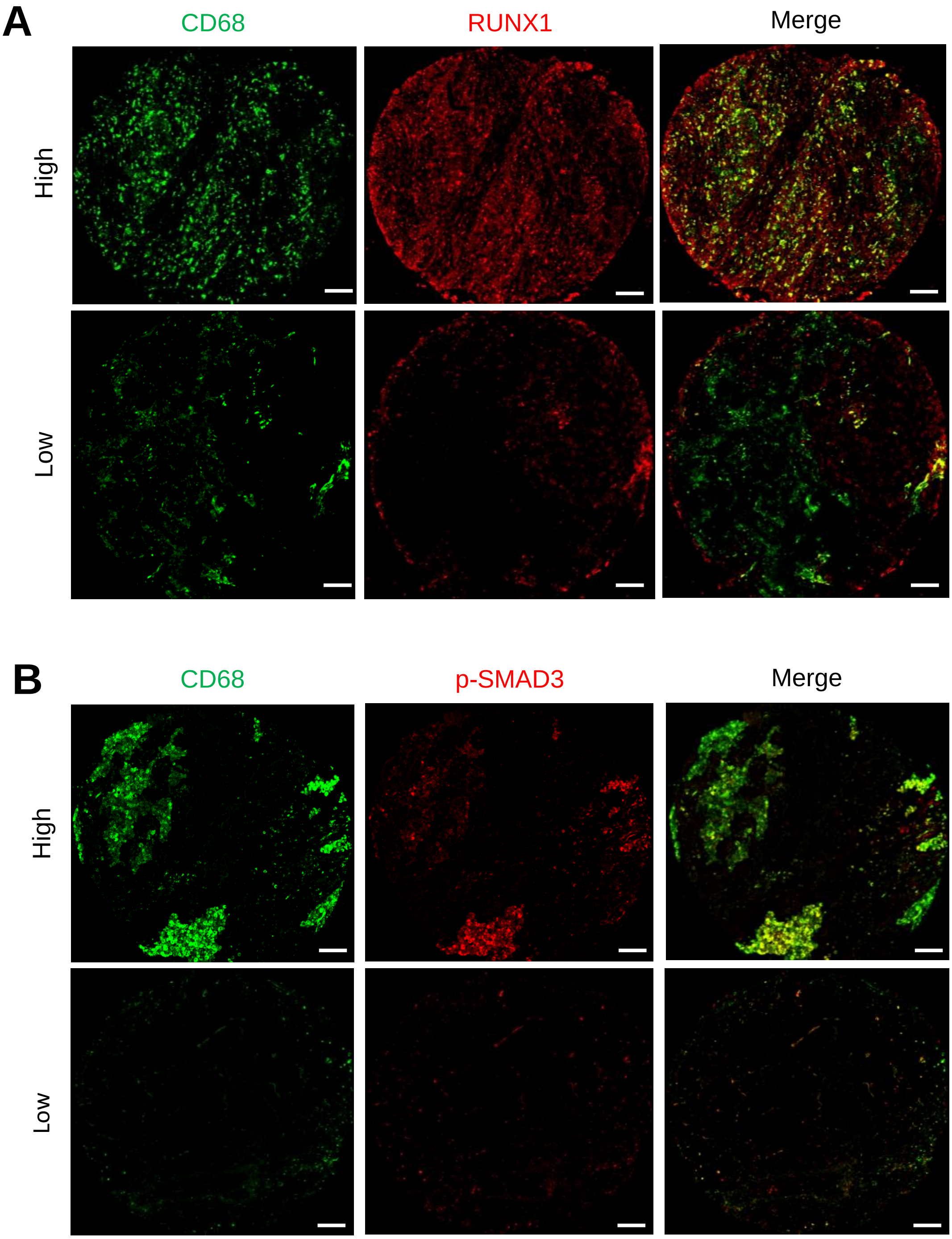

Supplementary Figure S2. Representative images of RUNX1 and p-SMAD3 expressions in TAMs showing by multiplex IHC staining for lung adenocarcinoma cohort. The samples of high and low expression levels of (**A**) RUNX1 or (**B**) p-SMAD3 in TAMs in the NSCLC tissue microarray were shown, where double positive cells were visualized in yellow. Scale bar, 100 μM.

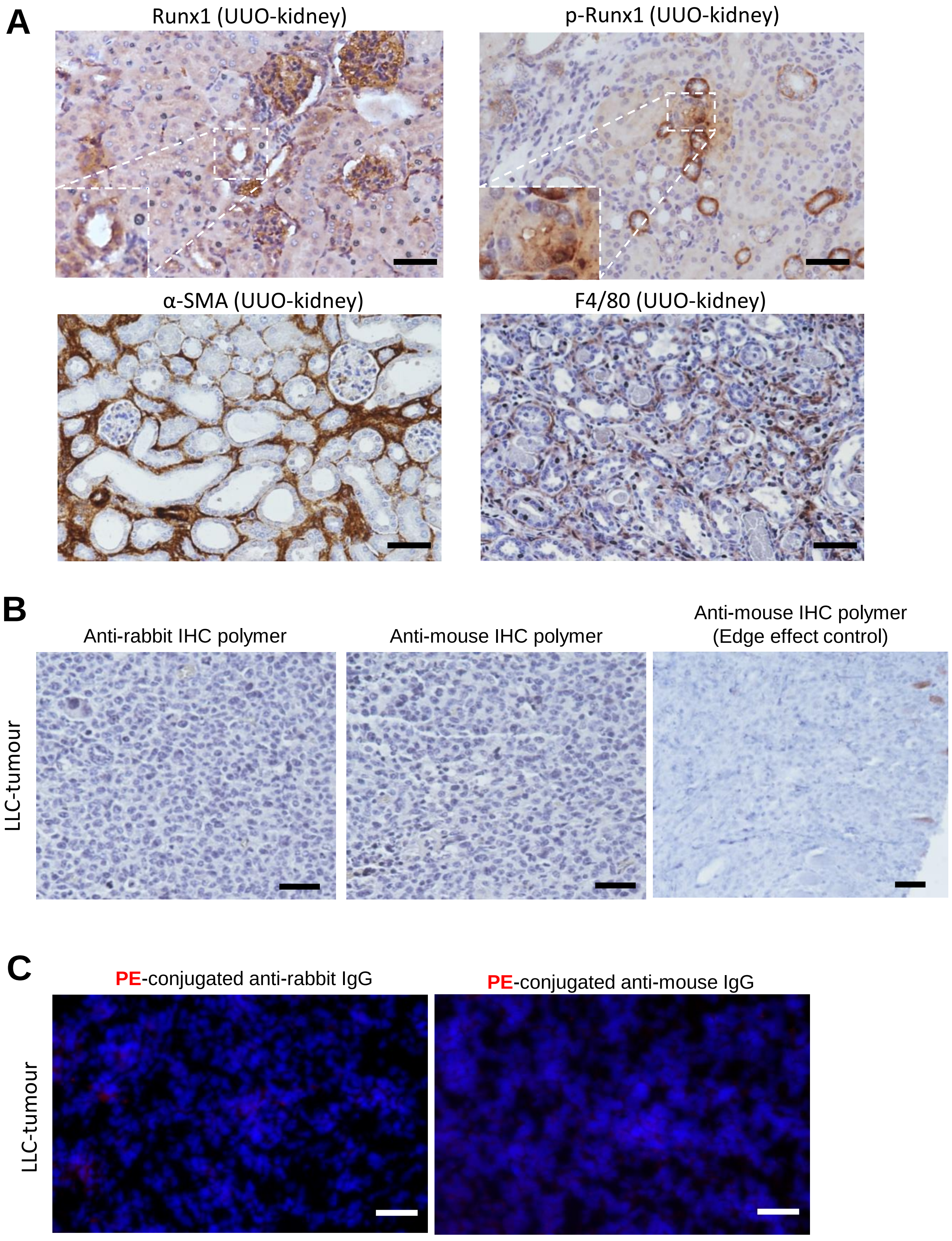

Supplementary Figure S3. Quality controls for immunostaining assays. (**A**) Positive controls for the IHC staining of Runx1, p-Runx1, α-SMA and F4/80 on the mouse fibrotic kidney injured by unilateral ureteral obstruction (UUO) [Tang et al, PNAS 2020]. (**B, C**) Negative controls ensured the absence of non-specific staining and edge effects in (**B**) IHC and (**C**) IF assays, showing on the mouse LLC-tumour samples stained with secondary antibody only. Scale bar, 50 μM

A

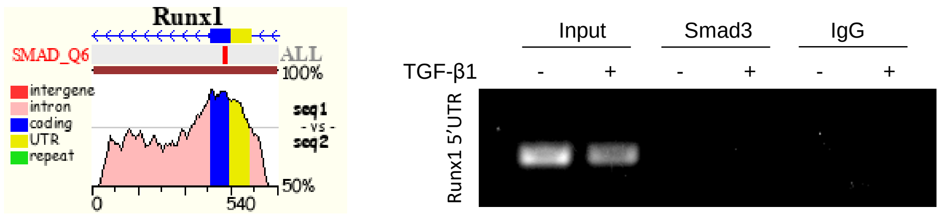

B

ATCTTAAAAAAAAAAAAAGTCAAACTCGATGACACTCAGGGTAGAGCTTCCCACAGCTGAG
GACTCTGGTTTGGGAACGCTGGTTAAGTTTGTGGCTGATCCTGTGGTTTGGTGGGAGTTGA
GCCGCAGTTGGGTCACACACACAGGCAGACTCGCACTCTTCGGA**AGTCAGCCA**CTGTCTA
CATTGGTTGAGTGAGTCAGCCCAGAGTCTGGCAAGATGATCTTCCTCCAACGTCGCCCGGC
GGTGGCTGGTTTTCTGCTGGGGCGCACAAATACCCACGAGCGAGCACCGTGTAACCGC

Supplementary Figure S4. Among two predicted binding sites on the 5'UTR region of Runx1 suggested by ECR browser, (**A**) Smad3 only binds to the conserved site in Figure 3G but not another one confirming by ChIP assay. (**B**) We further validated this finding by conducting dual-luciferase reporter assay, where Runx1 5'UTR expressing plasmids with or without the blue-highlighted Smad3 binding site was tested as shown in Figure 3K.

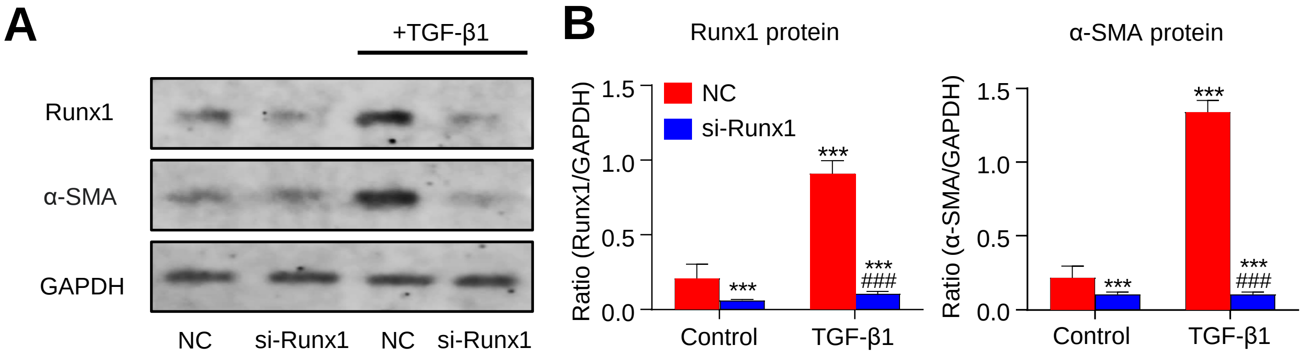

Supplementary Figure S5. Macrophage-specific Runx1 is important for MMT completion. (**A-B**) Western blot analysis showed that silencing of Runx1 effectively suppressed CAF marker (α-SMA) expression in BMDMs undergoing TGF-β1 driven MMT *in vitro* (n=5, ***P<0.001 vs NC-Control, ###P<0.001 vs NC-TGF-β1).

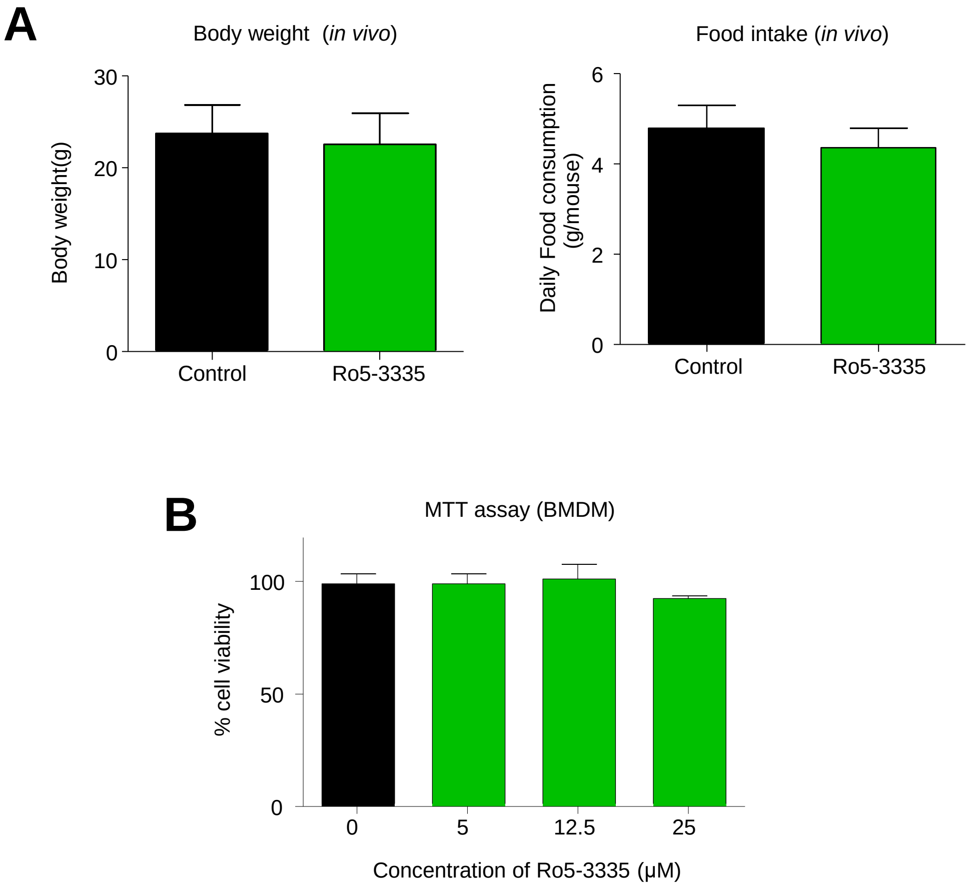

Supplementary Figure S6. Preclinical safety of Runx1 inhibitor Ro5-3335 *in vivo* and *in vitro*. (**A**) Ro5-3335 treatment (5 mg/kg/day) showed insignificant change in the body weight and food intake on the LLC-bearing mice in Figure 7. (**B**) In addition, no cytotoxic effect was observed in the Ro5-3335 treated BMDMs detected by MTT assay at 24h *in vitro* (n=5).

**Sequencing**	
Valid Barcodes	94.9%
Q30 (Barcode)	96.9%
Q30 (RNA Read)	94.7%
Q30 (RNA Read 2)	84.0%
Q30 (UMI)	96.4%
**Reads Mapped**	
Genome	75.4%
Confidently to Genome	74.0%
Confidently to Intergenic Regions	3.1%
Confidently to Intronic Regions	5.3%
Confidently to Exonic Regions	67.0%
Confidently to Transcriptome	63.8%
Antisense to Gene	2.1%
**Scrublet Doublet analysis**	
Doublet score < 0.4<br>(Genuine single cells population)	> 97%
Confidence value	>0.8 (high)

Supplementary Table S1. Quality control of the macrophage lineage scRNA-seq dataset.

	Forward	Reverse
Runx1	5’- AGCCTGGCAGTGTCAGAAGT	5’- CTTTCGAAAA-CGCACCTCTC
α-SMA	5’- GTGCTATGTCGCTCTGGACTTTGA	5’- ATGAAAGATGGCTGGAAGAGGGTC
FAP	5’- TTGACACCACCTACCCTCAC	5’- TACTCGTTCACTGGACACCC
GAPDH	5’-GCATGGCCTTCCGTGTTC,	5’- GATGTCATCATACTTGGCAGGTTT
Fn1	5’-TACCAAGGTCAATCCACACCCC	5’-CAGATGGCAAAAGAAAGCAGAGG
Col1a1	5’-TGCCGTGACCTCAAGATGTG	5’-CACAAGCGTGCTGTAGGTGA
Col1a2	5’-CAGAACATCACCTACCACTGCAA	5’-TTCAACATCGTTGGAACCCTG
Postn	5’-AAGCTGCGGCAAGACAAG	5’-GGGCTGTGTCAGGAGATCTTT
Hes1	5’-GTCCCTAGCCCACCTCTCTC	5’-AGGCGCAATCCAATATGAAC
Adam9	5’-GCGCTGTGTGGAAAGCTTC	5’-AAACACCGGCATGTCCTGTAC
Cd68	5’-CTTCCCACAGGCAGCACAG	5’-AATGATGAGAGGCAGCAAGAGG
F4/80	5’-TCTGGGGAGCTTACGATGGA	5’-GAATCCCGCAATGATGGCAC

Supplementary Table S2. Primer sequences for Real-Time PCR
